# Implementation pathways of a health services delivery redesign model to improve maternal and newborn outcomes in Kenya

**DOI:** 10.1136/bmjgh-2024-018240

**Published:** 2026-01-09

**Authors:** Meibin Chen, Tingting JI, Patrick T Wedlock, Victor Bwire, Everline Nyanchama, Jacinta Angote Mbelesia, Stephen Wandei, Anna Kalbarczyk, Kojo Nimako, Savitha Subramanian, David H Peters, Takeru Igusa, Olakunle Alonge

**Affiliations:** 1 Department of Civil and Systems Engineering, The Johns Hopkins University, Baltimore, Maryland, USA; 2 Department of Public Management, Harbin Institute of Technology, Harbin, People's Republic of China; 3 International Health, Johns Hopkins University Bloomberg School of Public Health, Baltimore, Maryland, USA; 4 Jacaranda Health, Nairobi, Kenya; 5 County Government of Kakamega, Kakamega, Kenya; 6 World Bank, Washington, District of Columbia, USA; 7 Bill and Melinda Gates Foundation, Seattle, Washington, USA; 8 Faculty of Health, York University, Toronto, Ontario, Canada; 9 Sparkman Center for Global Health, The University of Alabama at Birmingham, Birmingham, Alabama, USA

**Keywords:** Kenya, Delivery of Health Care, Health systems

## Abstract

Persistent high maternal and neonatal mortality rates in low- and middle-income countries (LMICs) call for system-level improvements in healthcare services. However, implementing such health system strengthening interventions presents challenges due to the complex, context-specific interactions inherent in these settings.

This paper presents implementation pathways of a service delivery redesign (SDR) model in Kakamega County, Kenya, offering insights into how complex health systems strengthening interventions can improve maternal and neonatal health (MNH) outcomes at scale in an LMIC setting. Drawing on a theory-of-change approach, key factors influencing the supply and demand of MNH services were identified and organised into a conceptual framework. Causal relationships were mapped through a participatory group model-building workshop into causal loop diagrams, and strategies were proposed to address barriers and facilitators to the SDR implementation process.

Several critical factors were identified along causal pathways as essential to implementation success. At the community level, building trust for expectant mothers in the health system reinforces use of quality services. Across facilities, having a well-functioning and efficient referral system ensures timely, coordinated multilevel care that improves patient outcomes. Between the facility and policy level, a delicate balance between meeting increased demand for services with available resources and available resources with supportive financial policies needs to be maintained. Across these system functions, trust emerges as a key factor initiating and reinforcing positive patterns. Prioritising efforts that encourage co-creation, ongoing coordination and engagement among relevant actors to build trust bolsters individual strategies (to increase demand, improve referral, build service readiness) and is key to improving MNH outcomes.

**Summary box**

- Service delivery redesign (SDR) has been proposed as a process of reorganising health services delivery systems to improve quality of maternal and neonatal health (MNH) services to reduce maternal and neonatal mortality.
- However, the implementation mechanism, that is, how and why SDR works or not, to inform strategic decision-making is not clear.
- This study shows that trust, and strategies for enabling trust, among actors across different health system levels act as a cross-cutting mechanism of SDR implementation success—reinforcing and sustaining critical factors such as demand for services, referral coordination, resource allocation and service readiness—to improve quality of MNH services.
- Upstream of trust, engagement with stakeholders is essential; it builds and sustains trust by fostering shared understanding and local buy-in in SDR, bolstering commitments and enabling resources and efforts to address any implementation challenges.
- Hence, efforts to implement SDR must be accompanied by intentional strategies to elicit trust among different actors; otherwise, SDR may not achieve its potential of improving quality of MNH services.

## Introduction

 High maternal and neonatal mortality rates continue to persist in low- and middle-income countries (LMICs) despite significant investments in health.^1^ Current global strategies to address maternal and neonatal health (MNH) in LMICs have primarily focused on increasing access and engagement with care services, such as enhancing antenatal care attendance and promoting facility deliveries.^2 3^ While these efforts have led to some reduction in maternal and neonatal mortality over the past two decades, their effectiveness has been hindered by poor quality of healthcare services.^4^ Notably, despite a steady increase in facility deliveries in recent years, the expected decline in mortality rates has not been commensurate.^2 5^

Recently, the Lancet Global Health Commission on High-Quality Health Systems proposed service delivery redesign (SDR) as a policy approach to improve MNH outcomes in LMICs.^4^ SDR is a form of health systems strengthening (HSS) intervention that involves the purposeful reorganisation of healthcare delivery processes to ensure that pregnant women receive the right care, at the right level of the system, and at the right time.^6^ In maternal health, SDR takes two main forms depending on context: one form restructures the system so that deliveries—especially high-risk cases—occur at well-equipped higher-level hospitals, as seen in Kenya^2 7^; the other strengthens primary care centres to handle deliveries, identify and manage complications with close coordination for referral to higher-level hospitals, as applied in settings such as Meghalaya State of India and Sindh province of Pakistan.^6^ These strategies often intersect with broader HSS components including health workforce deployment, governance, infrastructure and transportation.

Kakamega County in Kenya is facing high maternal and neonatal mortality rates, and <40% of births were delivered in hospitals with better MNH services.^2 7^ In Kakamega, SDR followed the first form: antenatal care was encouraged at lower-level or ‘spoke’ facilities (Levels 2 and 3), while deliveries were redirected to higher-level hospitals (Levels 4 and 5) designated as delivery ‘hubs’. This shift was simultaneously encouraged alongside staffing redistribution, emergency transportation strengthening, birth planning coordination and community mobilisation. A detailed description of the SDR model in Kakamega can be found in previous literature.^2 7^

Similar to other HSS interventions, SDR is a complex intervention involving multiple processes and various actors operating at different levels of the health system. The implementation pathways of SDR include the mechanisms and contextual factors through which the intervention is implemented in the health system and expected to be able to produce and improve MNH outcomes. Understanding these pathways through a systems lens is essential for identifying critical factors, uncovering potential barriers and facilitators and informing appropriate strategies to support effective implementation. Viewing SDR as operating within a complex adaptive system allows us to view the dynamics of its implementation in a structured holistic way, from tracing interactions among actors to detecting feedback loops and bottlenecks—while drawing on the contextual knowledge of stakeholders involved in implementation. Because HSS interventions are highly context-specific, insights from those with local experience across system levels are vital for tailoring interventions to real-world conditions. Without applying a systems lens, there is a risk of misapplying SDR, for example, shifting all deliveries to higher-level hospitals without ensuring quality care at those facilities or equitable access—which may result in overcrowding, disrespectful care, overmedicalisation and disparities in service access.^8^,^10^

The overarching objective of this paper is to identify the implementation pathways of the SDR model adopted in Kakamega County, with the aim of providing insights into how and why such intervention may succeed or fail in improving MNH outcomes at scale. Detailing implementation pathways alongside current strategies not only helps iterate on implementation processes of SDR in real-time but generates theories to understand the root causes of implementation success and failure.

To address this objective, we adopted a systems-oriented methodology with three main components: developing a conceptual framework to identify key factors influencing the supply and demand of MNH services relevant to SDR; creating causal loop diagrams (CLDs) through participatory group model-building workshops to map relationships and interactions across health system levels and identifying strategies that address facilitators and barriers to these factors from a systems perspective. The following section describes the methods used for these components. Then, the main results are presented as a conceptual framework, a set of CLDs representing the different SDR subsystems and a table of implementation strategies. In the discussion, we highlight the key implementation pathways that emerge from our findings, which may provide lessons for other settings seeking to implement similar SDR models,^11^ as well as for broader HSS interventions targeting MNH improvement in LMICs.

## Methods

The three components of our methodology are organised around three objectives, which taken together address the overarching aim of the study.

### Objective 1: identifying key factors influencing supply and demand of MNH services

To achieve the first objective, we employed a multistep, participatory approach to develop a conceptual framework that informs the implementation pathways of the SDR model in Kakamega. This process involved purposive engagement with stakeholders^12^ strategically involved in SDR design and implementation, systematic extraction of data from grey literature, and iterative synthesis of key concepts across supply and demand domains.^13^ The resulting framework maps system-level relationships and was organised using a theory-of-change^14^ approach and guided by the socio-ecological model.^15^ A detailed summary of the methodological steps is provided in table 1.

**Table 1:** Summary of steps for conceptual framework development

Step	Description
Stakeholder engagement	Purposively engaged stakeholders^12^ who played strategic roles in the design and implementation of SDR in Kakamega to identify grey literature Stakeholders included representatives from the local health system, implementation teams, non-government organisations and design consultants (online supplemental table A1 in Appendix) to bring diverse insights from policy, operational and design perspectives
Grey literature identification*	Materials were sourced from project documents, including proposals, protocols, operational procedures, budgets and workplans produced by various actors involved in the SDR initiative Six major documents were identified (online supplemental figure A1 in Appendix)
Data extraction	Extracted information under four categories: implementation process (eg, how MNH services were provided at different system levels),^38^ strategic objectives (eg, approaches for increasing supply and demand of MNH services), implementation outcomes (eg, fidelity and reach of implementation) and health outcomes (eg, morbidity/mortality)^39^ Performed by one coauthor and reviewed by a second coauthor
Conceptual aggregation	Aggregated extracted content into broader concepts,^13^ categorised under supply and demand domains (eg, ‘knowledge’ as a concept encompassing constructs such as awareness of care benefits and service locations) Conducted by one of the coauthors and reviewed by a second coauthor
Framework development	Linked concepts using a theory-of-change approach and in a logical sequence^14^ to represent pathways for improving MNH service use and delivery Relationships were further structured by system level using the socioecological model^15^ Reviewed by all coauthors.

* While this grey literature review approach ensured the relevance and contextual specificity of the documents collected—given that this particular SDR model has only been implemented in Kakamega—it may have excluded insights from other SDR variants or broader health systems strengthening efforts. However, expanding the scope to include such materials would likely exceed the focus of this study.

MNH, maternal and neonatal health; SDR, service delivery redesign.

### Objective 2: mapping causal relationships between key factors across health system levels

To achieve the second objective, we co-constructed CLDs with stakeholders to illustrate complex and dynamic relationships among key factors from the conceptual framework (eg, trust, use of quality antenatal, delivery and postnatal services, healthcare financing) and to demonstrate potential pathways—thereby suggesting strategies—through which these factors may interact to improve maternal and newborn health outcomes. CLDs, a tool from systems science, visually represent the directional relationships and feedback among system elements.^16 17^ They can be used to understand system behaviour by tracing how individual factor-to-factor relationships accumulate into broader pathways and can be developed iteratively with community partners to incorporate local knowledge into structured planning.^18^

The CLDs were created through group modelling and participatory activities over the course of a 2-day workshop in August 2021 in Kakamega with engagement from 50 different stakeholders. Stakeholders were purposively selected to ensure representation across policy, facility and community levels of the health system (online supplemental table A1 in Appendix), including county government officials, healthcare providers, pregnant women and community health workers. This multilevel stakeholder selection approach aimed to ensure a comprehensive understanding of the causal relationships and interactions shaping SDR implementation pathways from diverse perspectives.

Prior to the workshop, a set of initial CLDs was created, drawing from factors and relationships described in the conceptual framework. Four diagrams were created to reflect the main implementation levels for SDR in Kakamega: individual/household/community, spoke and hub facility, spoke/hub facility interface, and facility and policy interface.

During the workshop, participants were introduced to fundamental concepts for systems thinking and CLDs, a review of the conceptual framework, and the initial CLDs. The participants were then grouped based on the care delivery level (policy, hub facility (Levels 4 and 5), spoke facility (Levels 2 and 3) and community) that they represented, and the interfaces between these levels. Each group expanded on the initial CLDs, facilitated by a member of the research team with probing questions to identify the cause-effect relationships and feedback between factors.

Post workshop, the CLDs were cleaned and validated through an iterative process involving participants from the workshop and topic experts—focusing on drawing out causal loop relationships between factors discussed but not finalised during the sessions and obvious connections that were missed during the actual discussion.

### Objective 3: examining implementation strategies targeting barriers and facilitators

During the participatory group modelling workshop, and after the diagramming, an open discussion took place to critique and revise the CLDs as a large group. To achieve the third objective, research members guided the conversation toward identifying key facilitators and barriers—elements that either support or hinder the causal relationships—of critical linkages among the factors described in the CLDs.

Based on insights from the CLDs about ‘where’ and ‘how’ these facilitators and barriers can influence critical linkages along the implementation pathways, stakeholders proposed and implemented strategies in real-time to facilitate successful implementation—drawing from their experience implementing MNH services in the Kenyan context. Following the workshop, these strategies were coded using a published taxonomy of discrete implementation strategies and compiled in a table alongside their corresponding facilitators and barriers.^19^ These strategies were then organised according to the health system levels at which they operate or that they aim to influence, consistent with the structure of the conceptual framework.

## Results

The results of the three components of our methodology are presented below.

### Conceptual framework for the key factors of MNH services

**Figure 1:**
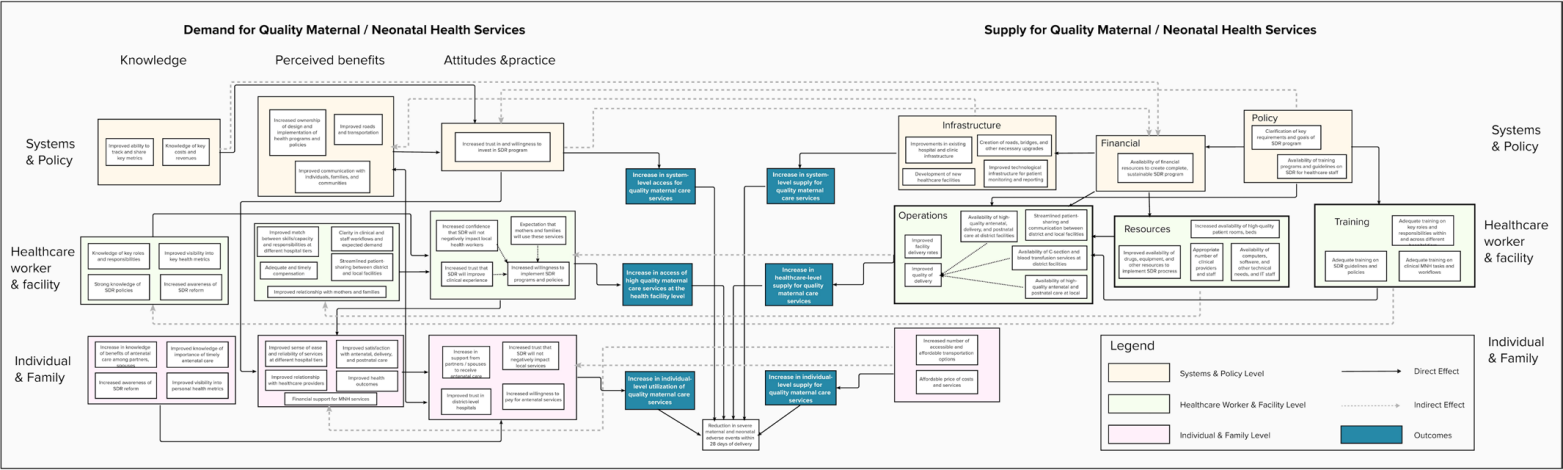
Conceptual framework of key factors of MNH services. MNH, maternal and neonatal health.

As shown in figure 1, the conceptual framework outlines three levels and two domains influencing MNH service delivery: system/policy, health facility/provider and family/individual levels under supply and demand domains. The framework highlights the significance of trust across multiple levels of the health system—policy, health worker and facility, and individual and family—as a key proximal determinant of access to and utilisation of high-quality maternal and newborn services under SDR on the demand side (online supplemental file 2). On the supply side, resource availability and service readiness further reinforce trust by building confidence in the health system’s capacity to deliver care (online supplemental file 3).

### CLDs showing causal relationships and interactions

**Figure 2:**
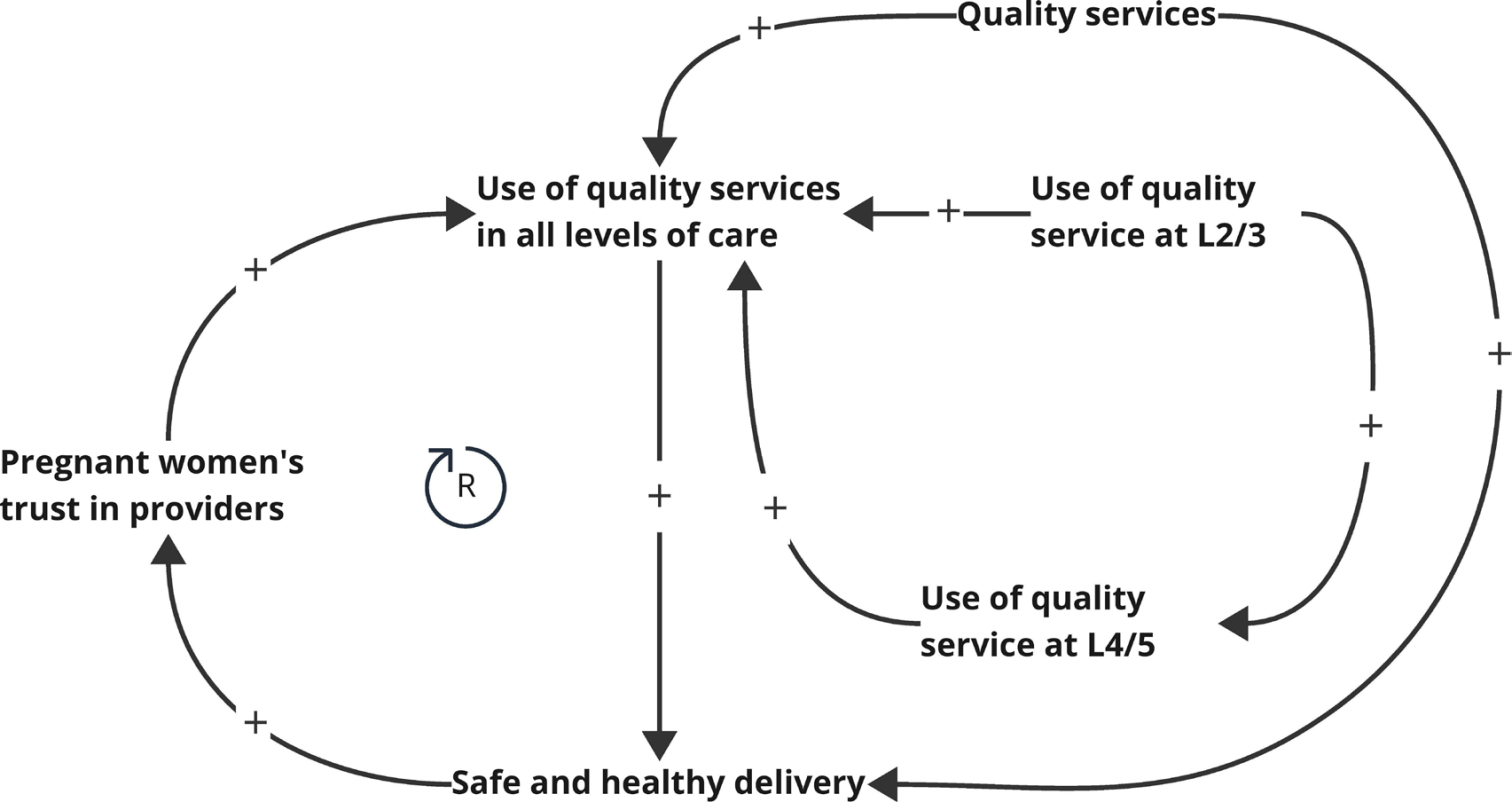
CLD of individual/household level (demand side). (**R**) refers to the reinforcing loop defined by the directed arrows connecting the factors immediately surrounding the symbol. In this figure, this loop describes how increased trust in providers leads to further use of quality services to positive MNH outcomes, which in turn continues to increase trust. CLD, causal loop diagram; MNH, maternal and neonatal health.

Four CLDs were developed to represent different levels of the system (figures2  3 and online supplemental figures A2 and A3 in appendix).

**Figure 3:**
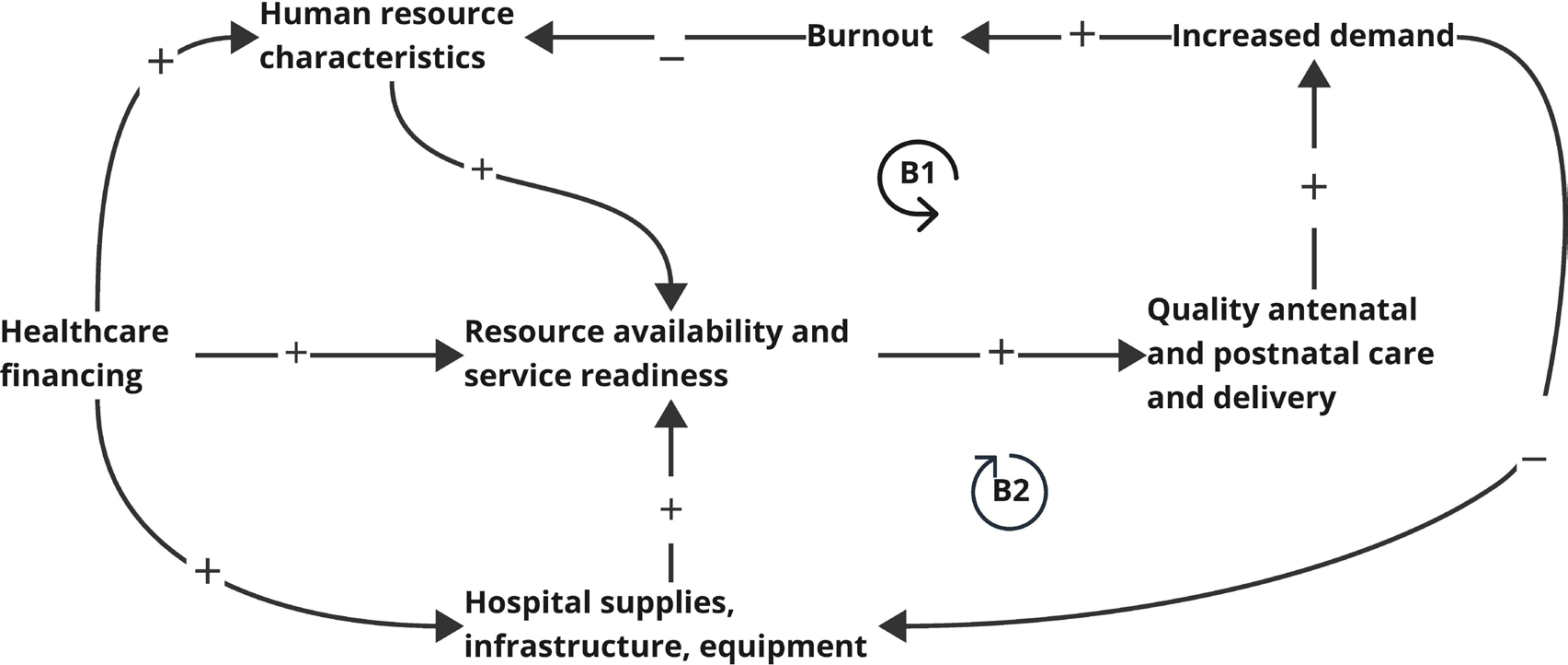
CLD of spoke/hub level facilities (supply side). (**B1**) and (**B2**) refer to the two balancing loops in this figure. The loops illustrate how human resources and equipment, infrastructure and supplies can increase quality care and demand for services. An increased demand for services can then deplete these resources, which is the balancing effect of these loops. The left part of the diagram shows how this trend can be prevented with responsive health financing. CLD, causal loop diagram.

Figure 2 illustrates demand-side dynamics at the community level. The main feature is the reinforcing loop (**R**) in which the use of quality services leads to positive MNH outcomes, which in turn strengthens expectant mothers’ trust in providers. This underscores the central role of trust-building in promoting pregnant women’ care-seeking for quality MNH services.

Figure 3 illustrates the supply-side dynamics at the facility level. Two balancing loops (**B1, B2**) show how increased demand for quality care can strain resources needed to sustain that quality. Supportive financial policies are therefore critical to prevent resource depletion and maintain service quality.

Online supplemental figure A2, which depicts referral dynamics, demonstrates how community trust initiates reinforcing loops (R1–R3): greater trust leads to more self-referrals and higher utilisation of quality ANC services. Effective referral and transportation systems further enhance outcomes, which then feed back into strengthening community trust (R4–R6).

Online supplemental figure A3, which depicts policy-level dynamics, shows how supportive policies can expand resource use across community and facility levels, thereby improving service readiness and MNH outcomes (R1, R2). These improved outcomes can, in turn, reinforce trust at both facility and policy level (R3, R4).

### Strategies, facilitators and barriers for key implementation pathways

A summary of strategies targeting key facilitators and barriers to SDR implementation is presented in online supplemental table A2 (appendix). The strategies span policy, facility and individual levels, illustrating how trust and confidence can be fostered as part of SDR across the health system to strengthen both the demand for and supply of quality MNH services.

## Discussion

This study identified key implementation pathways of an SDR model to improve MNH outcomes in Kakamega, Kenya, through participatory stakeholder activities and the application of system methods. It revealed that the success of a health services delivery redesign model like SDR depends on multifaceted and co-dependent strategies deployed contemporaneously at multiple health system levels. These strategies are in turn informed by a clear understanding of the complex non-linear relationships among salient factors along specific implementation pathways.

For SDR, trust emerged as a key factor—highlighted in the conceptual framework and further shown to initiate and reinforce positive feedback loops in the CLDs. While trust is only explicitly defined in two of our diagrams (figure 2 and figure A2 in online supplemental file 1), its influence spans all system levels: from trust of beneficiaries in care providers and policymakers, trust of care providers in health managers, to trust of health managers in policymakers. Trust acts in bi-directional ways to facilitate key system functions (eg, demand, resource availability, service readiness, referral and transportation) that determine the success of the SDR intervention. As noted in previous literature, when trust is present across levels, system actors are more willing to engage, coordinate and adapt to align the system towards better health system outcomes.^20^,^23^

Trust is universal and able to open efficient and effective pathways to optimise any level of resources and in any context for a better health system outcome—than when it is not present. Hence, strategies to directly build trust of beneficiaries, clients, care providers, health system managers and policy actors in SDR and its implementation are needed alongside other strategies that target demand, resource availability, service readiness, motivation of care providers and referral and transportation as part of SDR.

Hence, stakeholders’ engagement and participation at different health system levels and phases of intervention design and implementation was employed as the main direct strategy for building trust. Other strategies (to strengthen demand, resource availability, service readiness, motivation, referrals and transportation) also achieve their implementation and services delivery outcomes by eliciting confidence in various aspects of the implementation pathways and are therefore ‘indirect’ strategies for building trust—and must be considered as such in implementing SDR or any health system strengthening intervention.

The CLDs explicated the complex and dynamic relationships among different salient factors for implementing SDR successfully. First, by tracing causal pathways that lead to improved health outcomes, the CLDs help elucidate which relationships are part of patterns that support system performance. Second, within these positive pathways, critical linkages (where facilitators and barriers operate) emerge that pinpoint areas to target with both direct and ‘indirect’ strategies to build trust and confidence in the health system. These linkages represent strategic areas for intervention, where building and maintaining trust can amplify the success of intervention.

### Trust-building at the community level to promote pregnant mothers’ care-seeking for quality MNH services

At the community level, the CLD (figure 2) reveals a reinforcing feedback loop whereby utilisation of quality services that result in safe and positive delivery experiences increases the likelihood that women will continue to seek care within the health system. For successful implementation of the SDR model, pregnant mothers must be open to adopting the new delivery pathway, which encourages labour and delivery at higher-level referral facilities. However, for many women—particularly those who have not delivered at such facilities before or who may face greater geographical and financial barriers, there may be reluctance to transition to this model. To facilitate the use of quality services as depicted in the diagram, a mother must have initial trust that she will receive respectful, high-quality care at these higher-level facilities.

From our conversations, one strategy that emerged to build trust was through the deployment and empowerment of community healthcare workers (CHWs) (online supplemental table A2, appendix). As trusted members of the communities they serve, CHWs are well positioned to bridge gaps between the community and the health system.^3 20 22 24^ As highlighted earlier, trust operates across levels, through co-dependent strategies and in bi-directional ways. In this context, engaging CHWs not only builds trust for facility-based delivery, but also requires trust in CHWs by the community, and reciprocal trust between CHWs and the health system. Findings from Enguita-Fernández *et al*, in a similar sub-Saharan maternal health context, support this approach.^20^ They demonstrated that recruiting CHWs from within communities, involving local leaders and maintaining continuous two-way communication with the broader health system embedded CHW activities in local social dynamics and deepened community trust. While CHWs are a direct strategy to build trust among beneficiaries, ensuring trust in and of CHWs is essential to sustaining this implementation pathway.

### Trust-building between facility levels to ensure timely and responsive quality of care

CLD development revealed that a strong referral and transportation system is crucial in building community trust and ensuring timely and responsive care in LMIC contexts like Kakamega, where lower-level facilities often lack capacity to manage complications.^25^ Prior studies similarly emphasise the importance of referral services in matching care needs with available resources.^26^,^28^ During participatory modelling, stakeholders developed a dedicated CLD (online supplemental figure A2 in appendix) illustrating how referral and transfer processes shape maternal care-seeking behaviours and reinforce community trust between spoke and hub facilities.

The CLD identified reinforcing loops (R1, R2) showing how effective non-emergency referrals—such as taxis used by mothers for self-referral or inter-facility transfers—can facilitate timely access to higher-level care before complications arise. Improved maternal and neonatal outcomes from such referrals, in turn, reinforce community trust. For emergent cases, another reinforcing loop (R4) showed that rapid emergency transport to higher-level facilities reduces delays and further reinforces system responsiveness. In response, stakeholders proposed referral-related strategies (online supplemental table A2, appendix), including training healthcare workers on referral protocols and providing free transport for low-income mothers with complications.

### Trust-building at the facility/policy level to ensure resource availability and service readiness

Resource availability and service readiness, including essential supplies, infrastructure and a trained and motivated workforce, are critical for delivering high-quality MNH services.^29^,^33^ In figure 3, these factors are direct drivers for service quality and central to balancing between service demand and capacity (loops B1, B2). Stakeholders emphasised that without adequate investment and staffing, increased demand could lead to burnout and degraded care quality. To mitigate this, trust at both the facility and policy levels was identified as crucial—trust that financing will be sustained, policies will remain supportive and facilities will be equipped and staffed. The second CLD (figure A3 in online supplemental file 1) further illustrates how positive MNH outcomes can reinforce policy-level trust, prompting county-level investment through feedback loops (R1–R3). In this dynamic, supportive policy serves as a lever for sustained upstream and downstream resourcing.

Stakeholders proposed strategies to build and sustain trust at policy/facility level to ensure resource availability (online supplemental table A2, appendix), including policy engagement through local consensus meetings and mass media, and facility-level actions such as staff reallocation, clinical training, infrastructure investment and equipment procurement. These actions not only strengthen operational capacity but also reinforce institutional trust in the health system’s responsiveness.

### Stakeholder engagement for sustaining trust across multiple levels

The CLDs demonstrate how causal pathways leading to positive MNH outcomes hinge on critical linkages underpinned by trust—for instance, the assumption that quality services will be provided, the referral system will function reliably, and necessary supplies will be replenished when depleted. In an ideal implementation setting, these assumptions would hold. However, in practice, implementation is often iterative and marked by growing pains. While trust is needed to initiate a complex intervention like SDR, maintaining that trust throughout the implementation process is just as important.

One key strategy to reinforce trust over time is the continuous and structured engagement of stakeholders. Trust in the intervention and broader health system is more likely to be preserved when stakeholders, often with incomplete knowledge of the intervention, understand how specific activities contribute to outcomes, and how their localised experience and feedback are incorporated throughout the implementation process.^20 21 33^ Evidence from implementation science already highlights that meaningful stakeholder engagement enhances implementation capacity, improves uptake and increases the sustainability of health interventions.^34^,^36^

The use of CLDs sharpens this understanding. While trust has been described primarily as a key driver in critical linkages, CLDs also reveal where trust can break down—whether due to a poor neonatal outcome, resource constraints and coordination failures. When these breakdowns occur, stakeholder engagement plays a stabilising role, serving not only to rebuild trust but to maintain system momentum through uncertainty by maintaining alignment and promoting continuity. In this way, just as trust supports key implementation pathways, stakeholder engagement bolsters trust.

### Limitations

This study has some limitations. First, the grey literature review conducted prior to the stakeholder modelling exercise may have biased the CLDs toward strategies and factors mentioned in the reports. However, the goal of this study was to demonstrate the utility of a system method to explore the implementation of complex interventions within health systems. Moreover, the specific bias in this case was mitigated by involving a diverse group of stakeholders in the workshop, many of whom had not previously reviewed the literature. Second, the qualitative activities included a higher representation of supply-side participants compared with demand-side participants, potentially skewing the perspectives captured. As implementation continues with CHW engagement, the hope is for a better understanding of mothers’ and their families’ knowledge, perceived benefits, attitudes and practice in informing subsequent implementation phases. Finally, the qualitative activities were completed when implementation had commenced, which shaped the scope of feasible implementation strategies, as detailed in online supplemental table A2.

## Conclusion

This study examined the implementation pathways of an SDR model in Kakamega, Kenya, through participatory stakeholder activities and systems science methods. By mapping multilevel system dynamics via CLDs, the study identified critical factors and feedback loops that influence SDR implementation. A key finding was the central role of trust—as both an explicit and implicit mechanism—linking actors across system levels and reinforcing essential functions. Stakeholder engagement across levels and phases of implementation emerged as a direct strategy for building and sustaining trust throughout the system.

These findings may inform other sub-Saharan African contexts with similar health system structures. Other LMICs which face similar challenges like resource constraints and poor quality of care may consider applying systems methods and stakeholder engagement to improving systems functions. While specific implementation pathways will vary, this approach can help in identifying productive strategies and understanding implementation dynamics.

For next steps, insights from these activities will inform ongoing SDR adaptation in Kakamega. CLD insights—such as the importance of CHW engagement in increasing the trust of pregnant mothers, which will guide strategy prioritisation in subsequent phases. Additionally, the qualitative findings will inform the development of a quantitative systems model for evaluating different SDR implementation scenarios. This quantitative model will be useful for predicting the impact of SDR, other complex interventions and activities to strengthen health systems in Kenya and other LMICs.^37^

## Supplementary material

10.1136/bmjgh-2024-018240 online supplemental file 1

10.1136/bmjgh-2024-018240 online supplemental file 2

10.1136/bmjgh-2024-018240 online supplemental file 3

## Data Availability

All data relevant to the study are included in the article or uploaded as supplementary information.
