# Supplementary material for: Implementation pathways of a health services delivery redesign model to improve maternal and newborn outcomes in Kenya
Source: BMJ Glob Health. 2026 Jan 9;11(1):e018240. doi: 10.1136/bmjgh-2024-018240 (PMC12815182; doi:10.1136/bmjgh-2024-018240)
Supplement: online supplemental file 1 [file bmjgh-11-1-s001.docx]

Table A1. Stakeholders involved in identifying implementation pathways of SDR in Kakamega.

| Purpose | Stakeholders | Role |
| --- | --- | --- |
| Identify grey literature documents | *Local health system team*  - Kakamega County Health Team  *Implementation team*  - Jacaranda Health  - Rescue.co  *Design team*  - Thinkwell  - Mass Design  - Think Place | *Contributed to:*  Conceptualization, policy formulation, and coordination of health services  Implementation of SDR activities and resource mobilization  Implementation of SDR activities related to transfer and referral  Design of SDR model  Design of SDR model  Design of SDR model |
| Develop CLDs and identify barriers, facilitators, and strategies in workshops | *Policy-level stakeholders*  - Kakamega County government  - Jacaranda Health  - Bill and Melinda Gates Foundation  *Facility-level stakeholders*  - Healthcare representatives from the County  - Facility-in-charges from the different health facility levels  - Rescue.co  *Community-level stakeholders*  - Pregnant mothers  - Community healthcare workers | *Contributed to creation of:*  CLDs for policy-level and interface with hub facilities  All CLDs  CLDs for policy-level and interface with hub facilities  CLDs at the hub and spoke facilities, and interface with policy-level  CLDs at the hub and spoke facilities, and interface between hub and spoke  CLDs at the interface of hub and spoke facilities and community  CLDs at the hub and spoke facilities, and interface with community  CLDs at the hub and spoke facilities, and interface with community |

**Figure A1.** Grey Literature Documents Identified on the Implementation of MNH SDR Model in Kakamega County, Kenya.


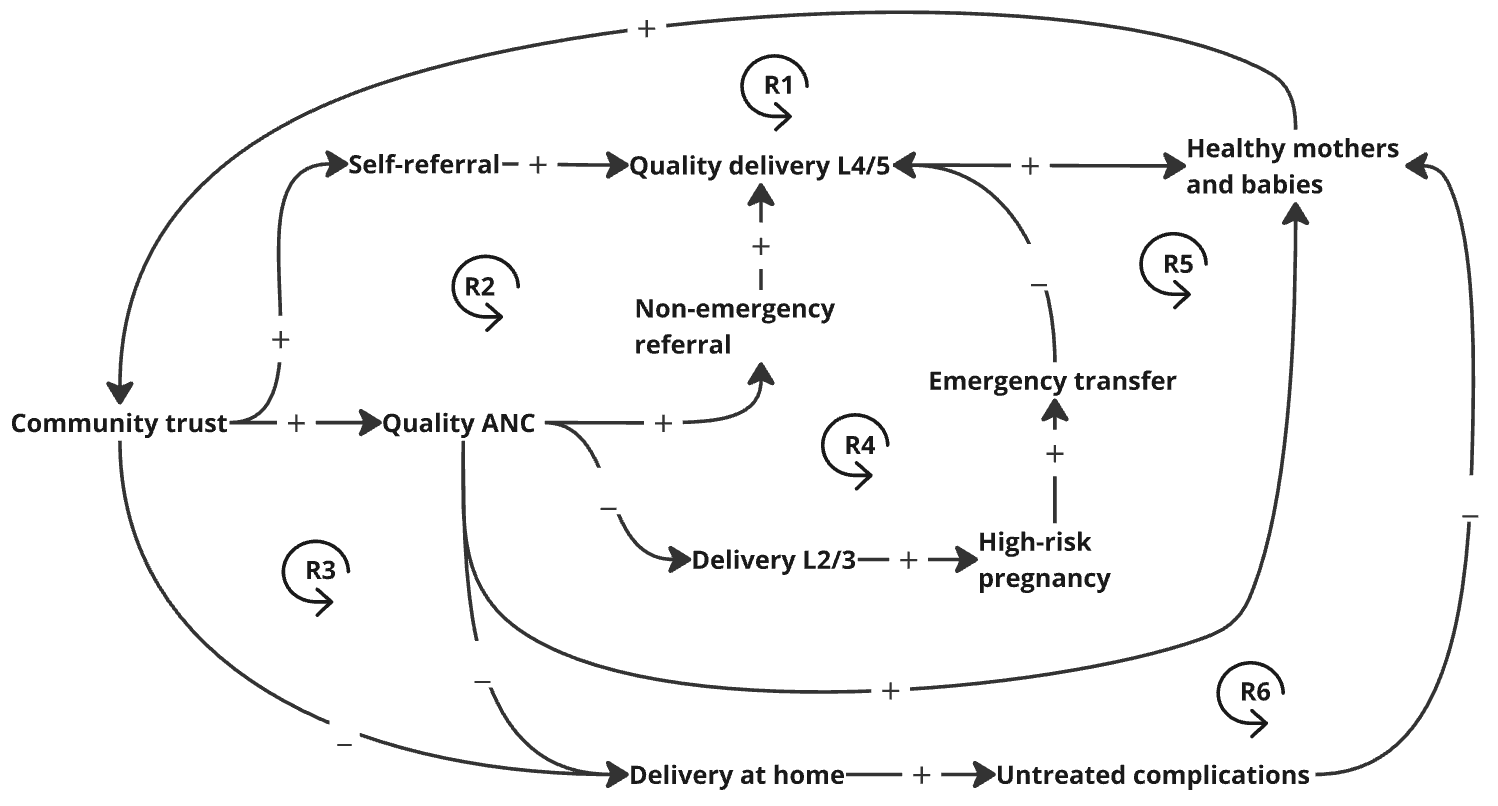


**Figure A.1** CLD of interface hub and spoke facilities.


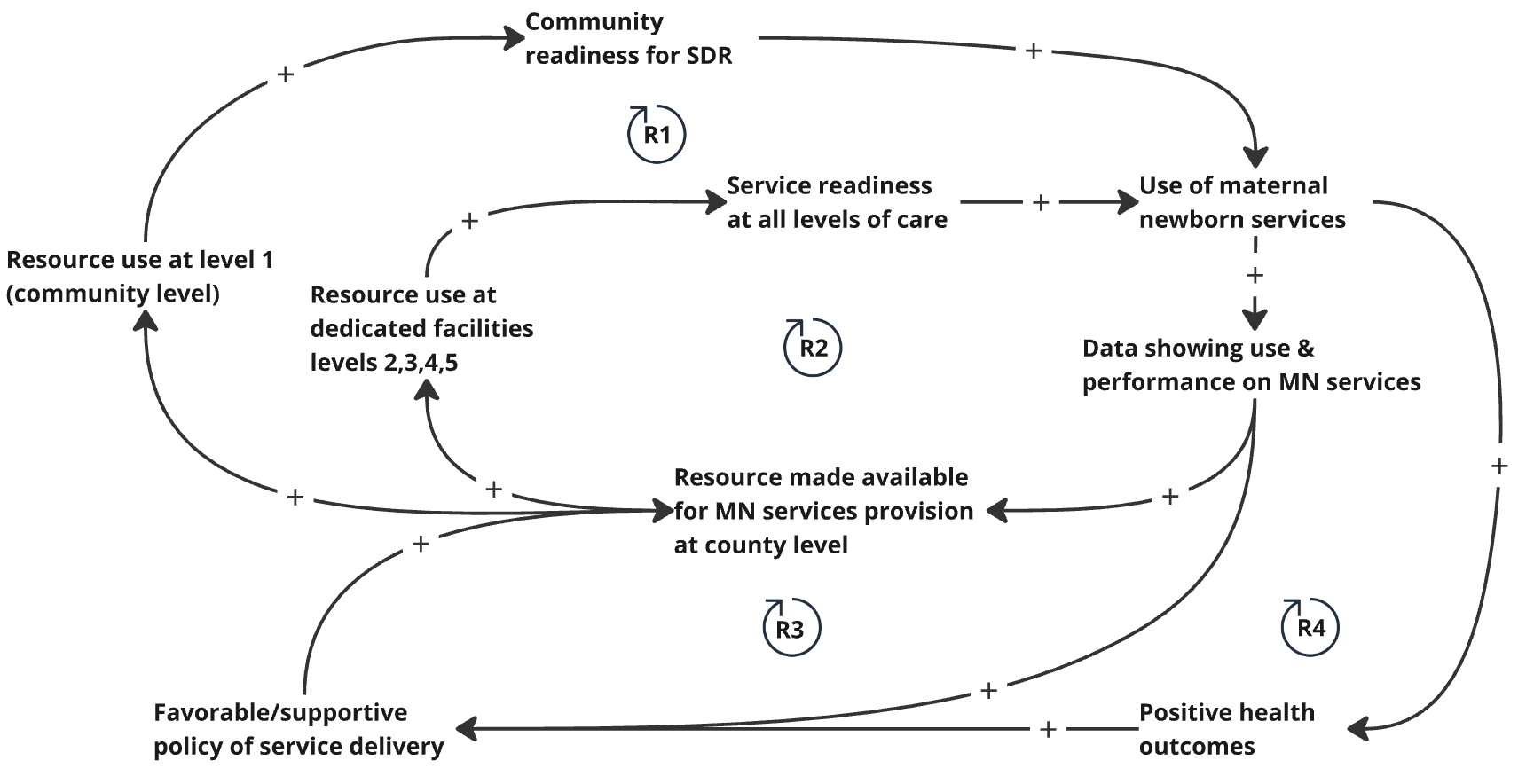


**Figure A.2** CLD of the interface of the hub/spoke and policy/system level.

Table A2. Summary of implementation strategies for trust-building across multiple levels targeting facilitators and barriers to SDR implementation.

|  | Trust-building Strategy | Facilitator | Barrier |
| --- | --- | --- | --- |
| Systems and Policy | | | |
| Demand for MNH services | - **Conduct local consensus discussions:** conversations and conceptual modeling workshops took place with local stakeholders - **Use mass media:** use media to spread the word about the SDR interventions | - Favorable/supportive policy environment around SDR - Well-engaged and involved stakeholders - Innovations & research - Advocacy & Media Influence | - Competing priorities - Bureaucracy |
| Supply of MNH services | - **Make billing easier:** additional financial support for clients without Linda Mama (a government-sponsored financial program used to subsize cost of care for qualifying beneficiaries) with hospital bills - **Champions:** advocate for policies that support RMNCH and encourage stakeholder participation in policy making | Financial support   - Strict adherence to RMNCH budgets   Policy   - Political good will - Supportive legislative frameworks and FIF policies | Financial support   - Inadequate financing of RMNCH facilities - Delayed disbursement and NHIF funds   Policy   - Poor policies (i.e. caps on hiring of HCW, common pool for finances) |
| Healthcare Worker and Facility | | | |
| Demand for MNH services | - **Revise professional roles:** change roles of traditional birth attendants to birth companions, shift healthcare workers at lower-level and higher-level facilities for increased match in skill and capacity needs - **Conduct ongoing training:** regular training for healthcare workers at facilities - **Incentives:** alter incentive structures or use capitated payments to increase staff motivation - **Train for leadership:** train leaders for SDR change effort | HR characteristics   - Adequate resource mobilization - Capacity building for HCW - Supportive supervision - Staff mentorship - Staff motivation (re-imbursement) - Team building activities - Bench marking from best performing facilities | HR characteristics   - Burn out - Increased referrals from lower-level facilities - Negative staff attitude - Poor leadership and governance |
| Supply for MNH services | - **Change physical structure and equipment:** purchase of large equipment, infrastructure investments such as the building of neonatal and maternal units | Hospital supplies, equipment & infrastructure   - Inter-ministerial collaboration - Efficient and short supply chains | Hospital supplies, equipment & infrastructure   - Inadequate supplies & resources (poor infrastructure, monopoly of suppliers) - Inadequate equipment |
| Individual and Family | | | |
| Demand for MNH services | - **Educate through peers:** use community healthcare workers to encourage higher level facility delivery - **Communication and engagement strategies:** make engagement, time to build trust, aligned expectations | - Address myths and misconceptions - Address ignorance about labor and delivery - Engage using peer influence | - Bad perception of the necessity for delivering at higher level facilities - Knowledge gap |
| Supply for MNH services | - **Reduce patient referral fees:** cover round-trip transportation costs from homes to facilities for delivery using referral service Rescue.co (a free emergency transportation service) | - Improve communication and coordination - Remove transportation constraints (i.e. fix roads, more emergency vehicles) - Streamline referral system (i.e. by risk, with respectful engagement) - Increased awareness of services | - Transport constraints (e.g., not enough emergency vehicles traveling between homes to facilities, and only one-way transportation services from homes to facilities) |

Note: MNH refers to maternal and neonatal health; HCW refers to health care workers; FIF refers to Financial Intermediary Fund; RMNCH refers to Reproductive, Maternal, Newborn, and Child Health. The implementation strategies described here were proposed from stakeholders’ engagement after the CLD group modeling activities.
