# Supplementary material for: Implementation pathways of a health services delivery redesign model to improve maternal and newborn outcomes in Kenya
Source: BMJ Glob Health. 2026 Jan 9;11(1):e018240. doi: 10.1136/bmjgh-2024-018240 (PMC12815182; doi:10.1136/bmjgh-2024-018240)
Supplement: online supplemental file 2 [file bmjgh-11-1-s002.pdf]

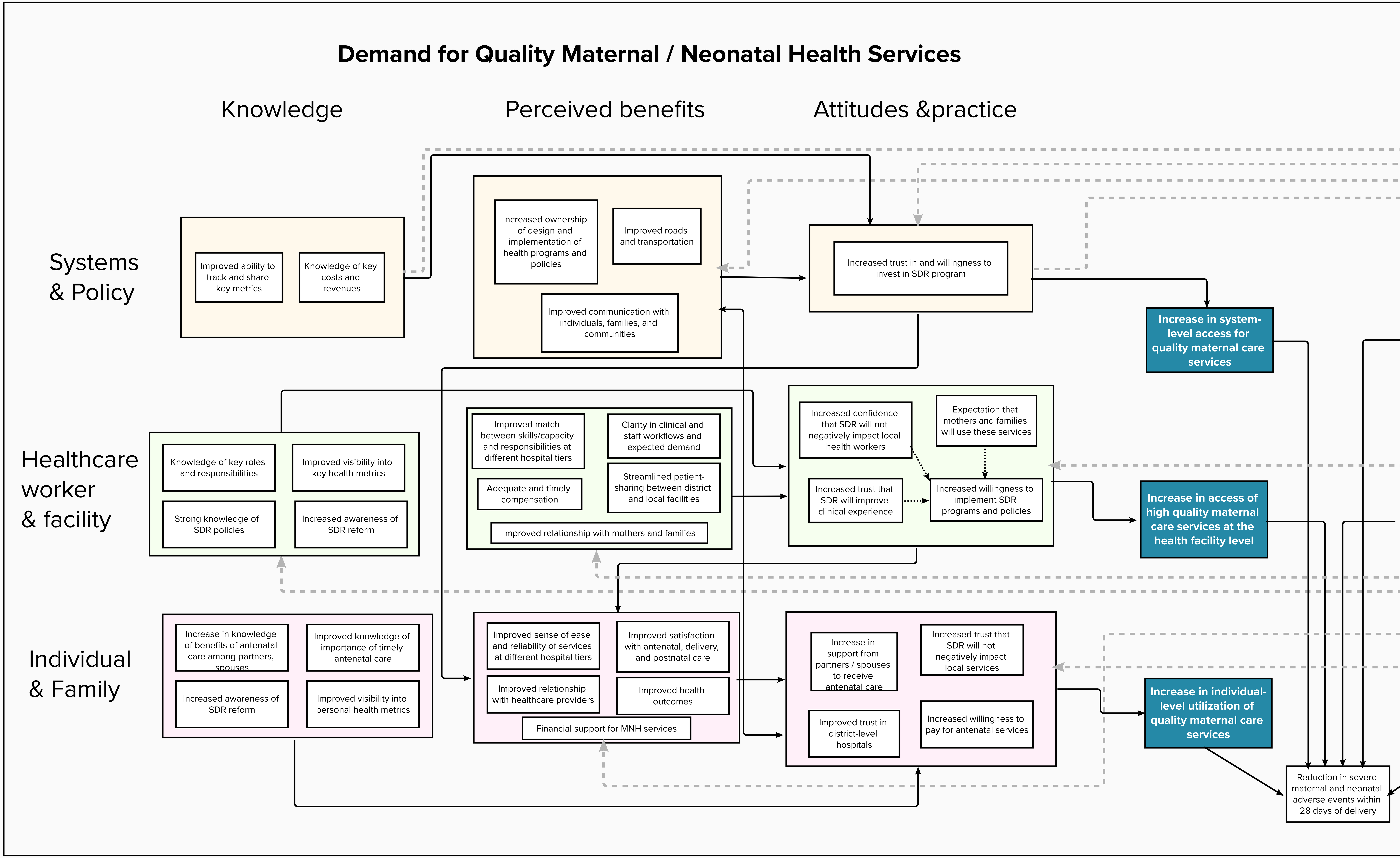
Demand for Quality Maternal / Neonatal Health Services
Knowledge
Perceived benefits
Attitudes &practice
Systems & Policy
Improved ability to track and share key metrics
Knowledge of key costs and revenues
Increased ownership of design and implementation of health programs and policies
Improved roads and transportation
Improved communication with individuals, families, and communities
Increased trust in and willingness to invest in SDR program
Increase in system-level access for quality maternal care services
Healthcare worker & facility
Knowledge of key roles and responsibilities
Improved visibility into key health metrics
Strong knowledge of SDR policies
Increased awareness of SDR reform
Improved match between skills/capacity and responsibilities at different hospital tiers
Clarity in clinical and staff workflows and expected demand
Adequate and timely compensation
Streamlined patient-sharing between district and local facilities
Improved relationship with mothers and families
Increased confidence that SDR will not negatively impact local health workers
Expectation that mothers and families will use these services
Increased trust that SDR will improve clinical experience
Increased willingness to implement SDR programs and policies
Increase in access of high quality maternal care services at the health facility level
Individual & Family
Increase in knowledge of benefits of antenatal care among partners, spouses
Improved knowledge of importance of timely antenatal care
Increased awareness of SDR reform
Improved visibility into personal health metrics
Improved sense of ease and reliability of services at different hospital tiers
Improved satisfaction with antenatal, delivery, and postnatal care
Improved relationship with healthcare providers
Improved health outcomes
Financial support for MNH services
Increase in support from partners / spouses to receive antenatal care
Increased trust that SDR will not negatively impact local services
Improved trust in district-level hospitals
Increased willingness to pay for antenatal services
Increase in individual-level utilization of quality maternal care services
Reduction in severe maternal and neonatal adverse events within 28 days of delivery
