# Supplementary figures and images for: Implementation pathways of a health services delivery redesign model to improve maternal and newborn outcomes in Kenya
Source: BMJ Glob Health. 2026 Jan 9;11(1):e018240. doi: 10.1136/bmjgh-2024-018240 (PMC12815182; doi:10.1136/bmjgh-2024-018240)

Supply for Quality Maternal / Neonatal Health Services

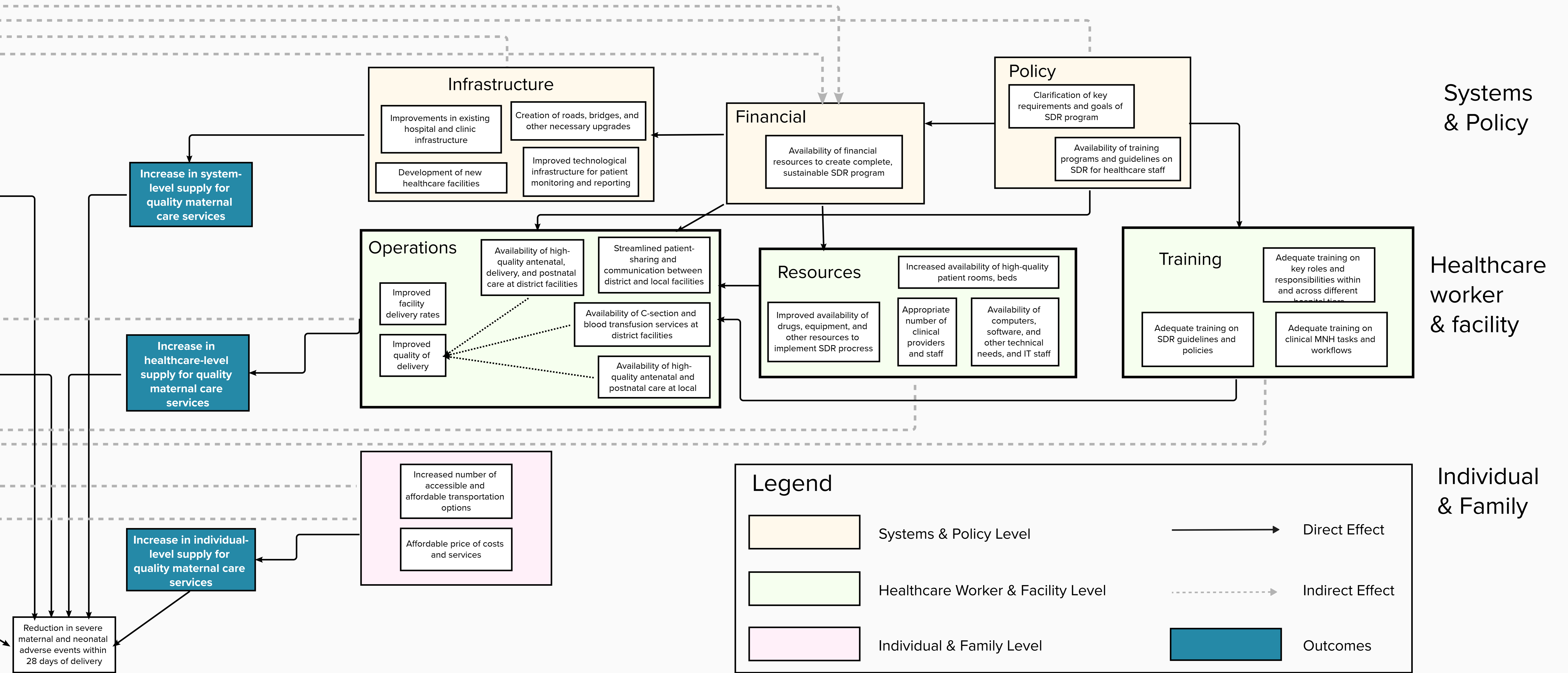

Supplement: online supplemental file 3 [file bmjgh-11-1-s003.pdf]
